# Target Genes of Autism Risk Loci in Brain Frontal Cortex

**DOI:** 10.3389/fgene.2019.00707

**Published:** 2019-08-09

**Authors:** Yan Sun, Xueming Yao, Michael E. March, Xinyi Meng, Junyi Li, Zhi Wei, Patrick M.A. Sleiman, Hakon Hakonarson, Qianghua Xia, Jin Li

**Affiliations:** ^1^Department of Cell Biology, 2011 Collaborative Innovation Center of Tianjin for Medical Epigenetics, Tianjin Key Laboratory of Medical Epigenetics, Tianjin Medical University, Tianjin, China; ^2^Center for Applied Genomics, The Children’s Hospital of Philadelphia, Philadelphia, PA, United States; ^3^College of Computing Sciences, New Jersey Institute of Technology, University Heights, Newark, NJ, United States; ^4^Division of Human Genetics, The Children’s Hospital of Philadelphia, Philadelphia, PA, United States; ^5^Department of Pediatrics, The Perelman School of Medicine, University of Pennsylvania, PA, United States

**Keywords:** autism, brain frontal cortex, DNA methylation, eQTL, GWAS loci, histone modification, target gene

## Abstract

Autism spectrum disorder (ASD) is a complex neuropsychiatric disorder. A number of genetic risk loci have been identified for ASD from genome-wide association studies (GWAS); however, their target genes in relevant tissues and cell types remain to be investigated. The frontal cortex is a key region in the human brain for communication and cognitive function. To identify risk genes contributing to potential dysfunction in the frontal cortex of ASD patients, we took an *in silico* approach integrating multi-omics data. We first found genes with expression in frontal cortex tissue that correlates with ASD risk loci by leveraging expression quantitative trait loci (eQTLs) information. Among these genes, we then identified 76 genes showing significant differential expression in the frontal cortex between ASD cases and controls in microarray datasets and further replicated four genes with RNA-seq data. Among the ASD GWAS single nucleotide polymorphisms (SNPs) correlating with the 76 genes, 20 overlap with histone marks and 40 are associated with gene methylation level. Thus, through multi-omics data analyses, we identified genes that may work as target genes of ASD risk loci in the brain frontal cortex.

## Introduction

Autism spectrum disorder (ASD) is a type of complex neurodevelopmental disorder mainly characterized by stereotyped behavior and deficiency in social communication ability. Among children, the prevalence rate of ASD has been estimated to be 1 in 68 in the USA and 1 in 100 worldwide, and there is four times higher prevalence among boys than girls (Ginsberg et al., 2012; Developmental Disabilities Monitoring Network Surveillance Year 2010 Principal Investigators; Centers for Disease Control and Prevention, 2014; Geschwind and State, 2015). ASD severely affects the life quality of patients and their families and increases public health burden (Ginsberg et al., 2013). ASD patients exhibit highly heterogeneous clinical presentations, and ASD patients are mainly treated by rehabilitation intervention with no specific therapeutic drug (Bowers et al., 2015). Therefore, it is necessary to understand the genetic mechanism underlying ASD development in important brain regions.

Genetic studies of ASD have revealed a number of risk loci that may contribute to ASD pathogenesis. It has been shown that single nucleotide polymorphisms (SNPs) located at loci 3p21 and 10q24, as well as in *CACNA1C* and *CACNB2*, are significantly associated with multiple psychiatric disorders including ASD (Cross-Disorder Group of the Psychiatric Genomics Consortium, 2013). Xia et al. discovered *TRIM33* and *NRAS-CSDE1* as ASD candidate genes by GWAS analysis of Chinese autistic patients and datasets of three European populations (Xia et al., 2014). A recent study by the Autism Spectrum Disorders Working Group of the Psychiatric Genomics Consortium (PGC) identified multiple loci, composed of common variants, associated with ASD and found a significant genetic correlation between ASD and schizophrenia *via* meta-analysis of more than 16,000 autistic patients (The Autism Spectrum Disorders Working Group of The Psychiatric Genomics Consortium, 2017). Furthermore, Cantor et al. found that rs289883 located in the intron of gene *PHB* was associated with the degree of behavioral abnormality in ASD patients (Cantor et al., 2018).

Different brain regions control different functions, which may be impaired in ASD patients. The frontal lobe of the brain plays an important role in social, emotional, and cognitive functions and has shown severe dysfunction in ASD patients (Courchesne and Pierce, 2005). The frontal lobes in ASD patients undergo an abnormal overgrowth while other regions are not significantly enlarged (Buxhoeveden et al., 2006). Additionally, a decrease in astrocyte precursor cells and an increase in synaptic connectivity are observed in the frontal cortex of ASD patients (Broek et al., 2014). Previous studies demonstrated pronounced ASD-associated gene expression changes in the cerebral cortex, including attenuated distinction between the frontal and temporal cortices in ASD brains (Voineagu et al., 2011).

Because of the importance of the frontal cortex in normal brains and its dysfunction in ASD brains, we aim to identify targeted genes of ASD risk loci in the frontal cortex. We obtained ASD associated SNPs from the GWAS catalog and found genes with genotype-correlated expression in the frontal cortex tissue from eQTL databases. By analyzing microarray gene expression datasets, we then identified 76 ASD–loci correlated genes showing significant expression difference between ASD brain frontal cortices and controls, and we further replicated four genes in an RNA-seq dataset. Among the ASD GWAS SNPs correlating with the 76 genes, 20 overlap with histone marks and 40 were associated with the gene methylation level, suggesting that they may regulate the transcription of their target genes through epigenetic mechanisms. Our results help to understand how ASD GWAS loci confer disease risk and prioritize genes for further functional validation.

## Material and Methods

### Extraction of ASD GWAS Loci

Significant ASD associations were downloaded from the GWAS catalog (https://www.ebi.ac.uk/gwas/) (Macarthur et al., 2017) using the keyword “autism spectrum disorder,” and SNP information was extracted from downloaded data. We did not apply any significance threshold when extracting ASD SNPs from the GWAS catalog.

### eQTL Analysis

Genes with expression in the frontal cortex that correlate with the genotypes of ASD SNPs were extracted from two eQTL databases: GTEx (https://gtexportal.org/home/) (GTEx Consortium, 2013) and Braineac (http://caprica.genetics.kcl.ac.uk/BRAINEAC/) (Ramasamy et al., 2014). Only associations with *P* < 0.05 were extracted.

### Analysis of Microarray Data

Series matrix files of two microarray datasets GSE28475 (Chow et al., 2012) and GSE28521 (Voineagu et al., 2011) that compare transcriptome data in human brain frontal cortex between ASD cases and controls were downloaded from the Gene Expression Omnibus (GEO) database (https://www.ncbi.nlm.nih.gov/geo/) (Barrett et al., 2013). There are 16 ASD cases and 16 controls in dataset GSE28521, and there are 52 cases and 61 controls in dataset GSE28475. Microarray data underwent quality control with log2 transformation and quantile normalization. Differential expression analysis was performed using the linear regression approach (Limma model) and further meta-analyzed using Fisher’s method in the NetworkAnalyst portal (http://www.networkanalyst.ca/) (Xia et al., 2015) with adjustment for batch effects.

### Analysis of RNA-Seq Data

The SRA file of an RNA-seq dataset (GSE102741) (Wright et al., 2017), which similarly compares transcriptome data in human brain frontal cortex between ASD cases and controls, was downloaded from the GEO database. Dataset GSE102741 contains 13 ASD cases and 39 controls. The quality of RNA-seq raw reads was examined using FastQC (Andrews, 2010), and reads were aligned to the human reference genome (GRCh37) using software HISAT2 (Kim et al., 2015). Then transcripts were assembled and quantified using Stringtie (Pertea et al., 2015) with the reference gene annotation (GRCh37) as a guide. Differential expression analysis between cases and controls was conducted using edgeR (Robinson et al., 2010).

### Protein–Protein Interaction Network Analysis

The gene symbols were input into the NetworkAnalyst web portal, which maps each gene to protein–protein interaction (PPI) databases to construct networks. The PPI network was constructed among the 76 genes without further extension, with InnateDB (Breuer et al., 2013) as the source of protein interactions.

### Pathway Enrichment Analysis

We input the 76 genes into DAVID (https://david.ncifcrf.gov/home.jsp) (Huang Da et al., 2009) and PANTHER (http://www.pantherdb.org/) (Mi and Thomas, 2009) web portals for pathway analysis. The pathway databases used in our analyses are KEGG (Kyoto Encyclopedia of Genes and Genomes) (Kanehisa and Goto, 2000) and Reactome (Fabregat et al., 2017), respectively.

### Analysis of Methylation Data

The generation of genome-wide methylation profiles of 843 subjects on the Infinium HumanMethylation450 BeadChip by the Center for Applied Genomics, the Children’s Hospital of Philadelphia, was reported in a previous publication (Van Ingen et al., 2016). The methylation level of each methylation probe was represented by the *M*-values (the log2 ratio between the methylated and unmethylated probe intensities). The association of ASD GWAS SNPs with methylation probes in each of the 76 genes was assessed in a linear regression model including gender, age, and 10 genotype-derived principle components as covariates.

### Hi-C Data Visualization

We conducted Hi-C data visualization for the ASD loci and target genes through the 3D Genome browser (promoter.bx.psu.edu/hi-c/) (Wang et al., 2018) and the FUMA GWAS site (http://fuma.ctglab.nl/) (Watanabe et al., 2017) using reported brain Hi-C data (Schmitt et al., 2016; Won et al., 2016).

## Results

As the frontal cortex is involved in important brain functions, which are severely impaired among ASD patients (communication, language, social behavior, and complex cognitive functions), we are interested in identifying target genes of ASD risk loci in the frontal cortex region. To do this, we first extracted all reported ASD associated risk loci from the GWAS catalog. A total of 466 SNPs from 19 studies were extracted, with the highest reported association *P*-value of 1 × 10^−5^. The majority (97%) of these SNPs were located in non-coding regions. As these non-coding SNPs could function by regulating downstream target gene expression, we examined their potential regulatory effects in two eQTL databases [brain—frontal cortex tissue in the GTEx database (GTEx Consortium, 2013) and frontal cortex in the Braineac database (Ramasamy et al., 2014)].We found 457 genes from GTEx and 1,848 genes from Braineac with mRNA level correlated with the additive genotype of ASD GWAS SNPs (nominal *P* < 0.05). As GWAS loci and their targeted genes may not exhibit highly significant correlations in eQTL analysis, we took this less stringent threshold and combined the ASD–loci targeted genes from the two datasets, yielding a list of 2,098 genes. The eQTL associations suggest that the expression of these genes may be directly or indirectly influenced by the genotype of the ASD loci.

We hypothesized that the expression of genes functioning in the frontal cortex may be dis-regulated among ASD patients; thus, we conducted gene expression meta-analysis by comparing the mRNA level of ASD cases and that of healthy controls at the genome-wide scale using datasets GSE28475 and GSE28521 from the GEO database. The analysis yielded 893 differentially expressed genes (adjusted *P* < 0.05). Among the 2,098 genes likely regulated by ASD–loci, 76 displayed significant differential gene expression (**Supplementary Table 1**), implicating that these genes may be ASD loci-controlled genes in the frontal cortex.

As replication, we looked into ASD RNA-seq dataset GSE102741 in the GEO database. We similarly conducted transcriptome profiling analysis and found that four of the 76 genes identified in the above steps showed significant differences (*P* < 0.05) in mRNA level between ASD cases and healthy controls: *HIST1H1C*, *HSPA1B*, *PRPF3*, and *SERPINA3* (**Table 1**). Therefore, differential expression of these four genes in the frontal cortex of ASD brains were further validated by RNA-seq. Inability to validate the remaining 72 genes could be due to the small sample size of the RNA-seq dataset. We further checked brain Hi-C data and found additional supporting evidence for the plausible chromatin interactions between ASD SNPs and the target genes (**Supplementary Figure 1**). Certainly, future Hi-C experiments specifically focusing on the frontal cortex regions should be performed to examine these interactions. It has been reported in the human protein atlas database (https://www.proteinatlas.org/) (Uhlen et al., 2015) that both the mRNA and proteins of ASD target genes *HIST1H1C* and *PRPF3* were detected in human brain cerebral cortex; HIST1H1C protein level is particularly high. The HSPA1B protein and SERPINA3 mRNA were also detected in the cerebral cortex. By searching the Mouse Genome Informatics (MGI) database (http://www.informatics.jax.org) (Law and Shaw, 2018), we also found that mRNA of mouse HIST1H1C and PRPF3 homologues has been detected in mouse brains in previous publications (Mckee et al., 2005; Diez-Roux et al., 2011). Furthermore, abnormal behavior, neurological phenotype, or defects in nervous system have been documented for mouse strains carrying mutant Prpf3 or Hspa1b genes (Law and Shaw, 2018), suggesting the biological relevance of these genes to ASD.

**Table 1:** Candidate autism spectrum disorder (ASD) target genes that showed significant differential expression in both microarray and RNA-seq datasets.

ASD SNP	Target gene	eQTL *P*-value	Microarray *P*-value	RNA-seq *P*-value
rs75782365	*HIST1H1C*	0.0015	0.0316	0.0228
rs3132581	*HSPA1B*	0.0058	0.0121	0.00299
rs11587682	*PRPF3*	0.017	0.0145	0.0325
rs4905226	*SERPINA3*	0.014	0.0376	0.00629

SNP, single nucleotide polymorphism; Target Gene, candidate target gene identified for each ASD GWAS locus; eQTL P-value, P-value of correlation between gene expression level and GWAS SNP genotype in GTEx database or Braineac database; Microarray P-value, P-value of the differentially expressed gene in microarray meta-analysis; RNA-seq P-value, P-value of the differentially expressed gene in RNA-seq analysis.

**Figure 1:**
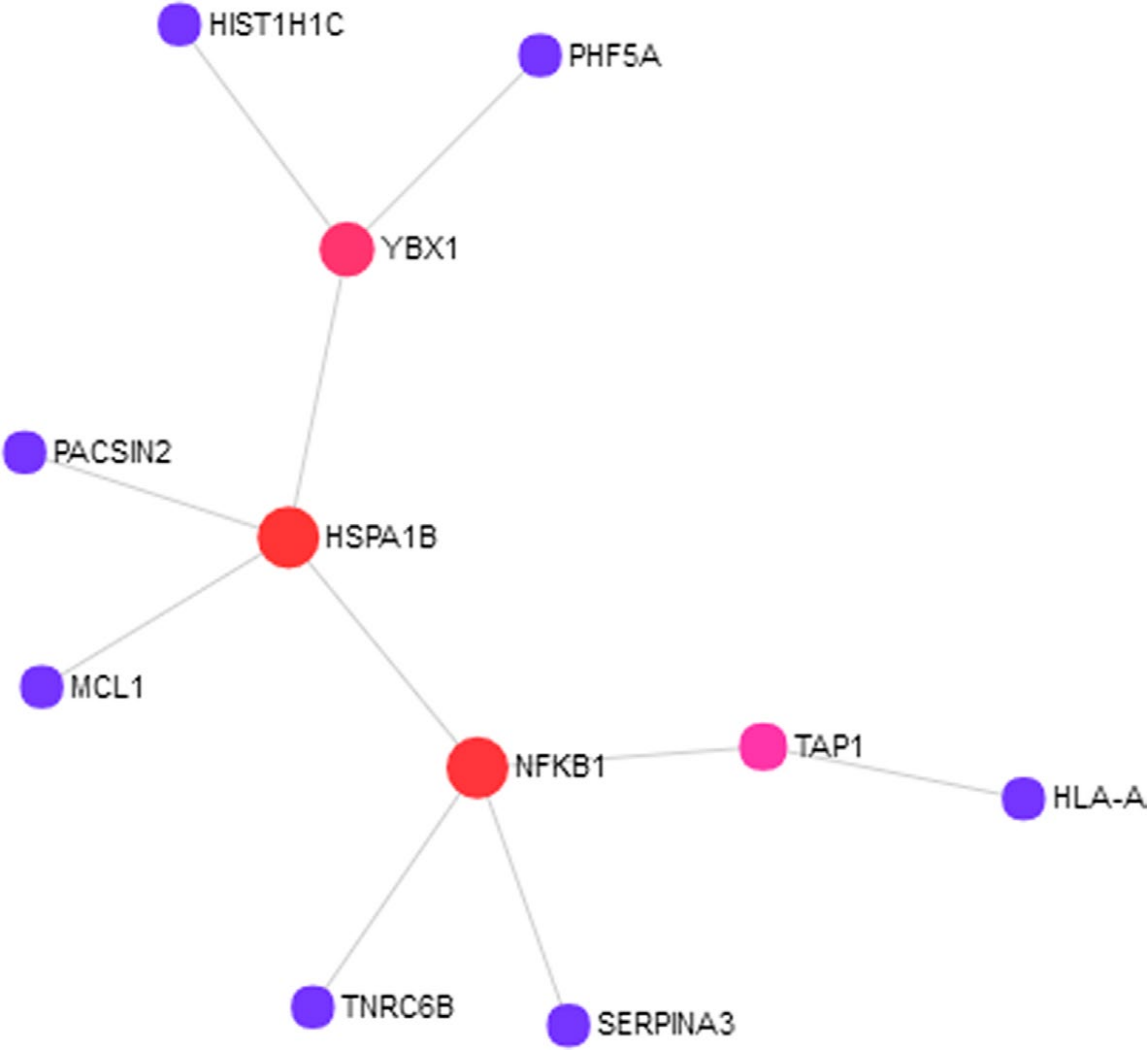
Eleven out of 76 proteins encoded by the autism spectrum disorder (ASD) loci target genes showed protein–protein interactions (PPIs). The PPI network was constructed using NetworkAnalyst with InnateDB as the source of protein interaction data. The nodes represent proteins, and the size of the nodes reflects the number of interaction partners with it. The edges between the nodes indicate known interactions between the proteins.

To understand how the 76 genes are involved in ASD pathogenesis, we constructed a protein–protein interaction (PPI) network (**Figure 1**) using NetworkAnalyst. The largest module consists of 11 of the 76 genes, including three of the RNA-seq validated genes (*HIST1H1C*, *HSPA1B*, and *SERPINA3*). To fully understand the interactions between these genes, we further examined the pathways in which these genes are enriched. We found significantly enriched pathways: “Antigen processing and presentation“ and “Noncanonical activation of NOTCH3“ (**Supplementary Table 2**). Both of these pathways are highly relevant to ASD pathogenesis (Needleman and Mcallister, 2012; Hormozdiari et al., 2015; Bennabi et al., 2018; Jones et al., 2018).

Both phenotypic and genetic overlap was observed between neuropsychiatric diseases. We found that SNPs in genes *HISTI1H1C*, *HSPA1B*, *PRPF3*, and *SERPINA3* showed at least nominal significant association with four other neuropsychiatric diseases [schizophrenia (SCZ), bipolar disorder, major depressive disorder (MDD), and attention-deficit hyperactivity disorder (ADHD)] in the Broad PGC database (https://data.broadinstitute.org/mpg/ricopili/) (Ripke and Thomas, 2017) (**Table 2**).

**Table 2:** Within genes *HISTI1H1C*, *HSPA1B*, *PRPF3*, and *SERPINA3*, single-nucleotide polymorphisms (SNPs) are associated with other neuropsychiatric diseases.

	ADHD		Bipolar		MDD		SCZ	
Gene	Best SNP	P-value	Best SNP	P-value	Best SNP	P-value	Best SNP	P-value
*HIST1H1C*	rs16891264	0.0529	rs10425	0.0022	rs12210098	0.0024	rs3857546	1.46E−09
*HSPA1B*	rs9267786	0.0202	rs389883	0.000322	rs9368699	0.00562	rs1270942	5.00E−07
*PRPF3*	rs10494266	0.00081	rs12138453	0.000171	rs16836940	0.00014	rs16835254	0.000105
*SERPINA3*	rs2268336	0.00107	rs17091191	0.00103	rs1243533	0.0162	rs11625527	0.00403

ADHD, attention-deficit hyperactivity disorder; MDD, major depressive disorder; SCZ, schizophrenia.

To understand how ASD SNPs may regulate the expression of their target genes, we explored the functional annotations of ASD GWAS SNPs corresponding to the 76 target genes in the ENCODE (Encode Project Consortium, 2012) and ROADMAP (Roadmap Epigenomics Consortium et al., 2015) epigenome databases *via* the HaploReg web portal (Ward and Kellis, 2016). We found 20 ASD SNPs overlap with histone marks in the brain dorsolateral prefrontal cortex (**Table 3**). This suggests that these SNPs may affect chromatin activation through histone methylation and acetylation, which in turn affects their target gene expression.

**Table 3:** ASD genome-wide association studies (GWAS) SNPs overlap with histone marks in brain dorsolateral prefrontal cortex, based on ENCODE and ROADMAP datasets.

SNP	Chromatin marks			
rs1080500		H3K4me1	H3K27ac	
rs1104918		H3K4me1	H3K27ac	
rs12045323		H3K4me1	H3K27ac	
rs12826178		H3K4me1		
rs133047		H3K4me1		
rs1550976		H3K4me1		
rs169738	H3K4me3	H3K4me1	H3K27ac	
rs17292804	H3K4me3	H3K4me1	H3K27ac	H3K9ac
rs2021722		H3K4me1	H3K27ac	H3K9ac
rs2233375			H3K27ac	
rs2297909		H3K4me1	H3K27ac	H3K9ac
rs2898883			H3K27ac	H3K9ac
rs4150167		H3K4me1	H3K27ac	
rs4702	H3K4me3	H3K4me1	H3K27ac	H3K9ac
rs548181	H3K4me3	H3K4me1	H3K27ac	H3K9ac
rs609412		H3K4me1		
rs73416724	H3K4me3	H3K4me1	H3K27ac	H3K9ac
rs760648		H3K4me1		
rs8321		H3K4me1		H3K9ac
rs880446	H3K4me3	H3K4me1	H3K27ac	H3K9ac

We also looked into whether there is any correlation between the genotypes of ASD SNPs and methylation at or near their target genes. Forty-five of the 76 genes contained probes with methylation level correlated with additive SNP genotype (**Table 4**) at a nominal significance level, suggesting that the expression level of these genes may be regulated by ASD SNPs through DNA methylation.

**Table 4:** ASD GWAS SNPs correlate with target gene methylation level at nominal significance level.

eQTL_SNP	Gene	Methylation probe	Methylation beta	Methylation *P*-value
rs385492	*HLA-F*	cg09296453	−0.204	1.25E−16
rs12887734	*ZFYVE21*	cg01651570	0.33	9.61E−07
rs4650608	*IFI44*	cg07107453	−0.0964	3.93E−06
rs2021722	*HLA-A*	cg20408505	−0.0653	0.000391
rs8054556	*SEZ6L2*	cg09584855	0.0426	0.000398
rs7746199	*PGBD1*	cg20029652	0.0364	0.000721
rs1080500	*SELK*	cg02011912	0.0449	0.000883
rs11587682	*APH1A*	cg04633021	−0.0633	0.00107
rs880446	*TCEA2*	cg03946671	−0.141	0.00162
rs1550976	*NTM*	cg04307764	−0.0481	0.00208
rs4150167	*HSDL1*	cg08941639	−0.253	0.00223
rs2332700	*RGS6*	cg12963168	0.0372	0.00239
rs2535629	*ABHD14A*	cg09254361	0.0693	0.00261
rs221902	*MED6*	cg16339264	0.033	0.00265
rs11191419	*ACTR1A*	cg11453585	−0.0485	0.00329
rs11587682	*PRPF3*	cg04212235	0.106	0.00405
rs6538761	*LTA4H*	cg09725090	0.0222	0.00502
rs12871532	*LIG4*	cg20776540	−0.0244	0.00547
rs11587682	*MCL1*	cg17724175	0.108	0.00597
rs72934570	*WDR7*	cg24533565	−0.0718	0.00636
rs880446	*ZBTB46*	cg04630273	0.0518	0.00655
rs8009147	*ADSSL1*	cg21190363	0.0317	0.00854
rs8321	*ABCF1*	cg05385119	0.148	0.00896
rs1080500	*CACNA2D3*	cg09890989	0.0324	0.0116
rs4773054	*COL4A1*	cg02673355	0.0716	0.0118
rs609412	*LARS*	cg17175376	0.022	0.0128
rs169738	*NUDT3*	cg25055477	0.0178	0.0152
rs2851447	*HIP1R*	cg26682900	−0.0185	0.0167
rs548181	*SRPR*	cg27218829	−0.0302	0.0202
rs72687362	*ARC*	cg08860119	−0.0361	0.0215
rs7914558	*SH3PXD2A*	cg07168060	0.0332	0.0217
rs2233375	*INPP5D*	cg12315466	0.034	0.0226
rs12826178	*SHMT2*	cg08163918	−0.133	0.025
rs548181	*HYLS1*	cg21050392	−0.0281	0.0281
rs10255295	*ZC3HAV1*	cg14341575	0.034	0.0287
rs1797052	*POLR3C*	cg19990379	−0.0364	0.0306
rs3849046	*REEP2*	cg07368507	0.0279	0.0308
rs1104918	*AEN*	cg21347380	−0.0318	0.0371
rs6453278	*AGGF1*	cg15817406	−0.0526	0.0384
rs7254215	*NOTCH3*	cg01282080	−0.0401	0.0388
rs7184114	*GPR56*	cg25645462	−0.0301	0.0396
rs11735612	*PCDH18*	cg06787716	−0.0219	0.0398
rs2898883	*SPOP*	cg08001899	0.0471	0.0412
rs133047	*XRCC6*	cg26919805	0.0557	0.0428
rs73416724	*MRPL2*	cg01847614	−0.0244	0.0456

## Discussion

To find ASD GWAS loci targeted genes, we conducted an analysis integrating eQTL, transcriptome, epigenome, and methylation data. We began by analyzing the correlation between SNP genotype and mRNA level of genes reflected by eQTL data, and we obtained 2,098 target genes that may be regulated by these ASD loci. Then, we analyzed the differentially expressed genes between cases and controls at the mRNA level using array and RNA-seq data. A total of 76 genes with expression correlating with ASD SNP genotype were differentially expressed between ASD cases and controls in the frontal cortex in array data. Four of those genes were further validated by RNA-seq data. Evidence also suggested that the expression level of these genes could be regulated through histone modification or DNA methylation. Therefore, by *in silico* analysis, we identified candidate genes likely controlled by ASD loci in the frontal cortex, which are worthy of further experimental validation.

There are multiple lines of evidence suggesting the involvement of the four candidate ASD genes in disease etiology. *HIST1H1C* encodes a protein that belongs to the histone cluster 1 H1 family. In an ASD model system based on haploinsufficiency of *SHANK3*, Darville et al. found that five histone isoforms including HIST1H1C were down-regulated upon lithium and VPA treatment in neurons differentiated from pluripotent stem cells. Lithium and VPA increased levels of *SHANK3* mRNA, and the authors speculated that *SHANK3* may be regulated through an epigenetic mechanism involving histone modification (Darville et al., 2016). In addition, *HIST1H1C* may also be involved in other brain disease development. For example, *HIST1H1C* displayed consistently significant increased mRNA level in the cortex of brains from 7- and 18-month-old mice in an Alzheimer’s disease model (Ham et al., 2018). The mRNA level of *HIST1H1C* is up-regulated in hypoxia and is correlated with worse disease outcome among neuroblastoma patients (Applebaum et al., 2016). Mutation in other members of histone cluster 1 H1 family, such as HIST1H1E, has been detected in ASD patient and is likely to be the underlying causal mutation (Duffney et al., 2018). Systematic review indicated that nearly 20% of ASD candidate genes play a role in epigenetic regulations, especially histone modifications (Duffney et al., 2018). These data support the potential involvement of HIST1H1C in ASD development, likely through epigenetic regulation of neurodevelopmental genes.

*HSPA1B* encodes a heat shock protein, which works as chaperone for other proteins. In heat shock experiments on induced pluripotent stem cells modeling brain development under maternal fever, *HSPA1B* is one of the heat shock genes that drastically increased its mRNA level, together with other genes involved in neurogenesis and neuronal function (Lin et al., 2014). Heat shock proteins target mis-folded proteins and facilitate proper refolding or targeting of damaged proteins for degradation (Lin et al., 2014). Its mRNA level is increased in the frontal cortex of schizophrenia subjects (Arion et al., 2007). It has been shown that *HSPA1B* also functions in the pathogenesis of other neurological conditions, such as Parkinson disease (Kalia et al., 2010), progressive supranuclear palsy (Hauser et al., 2005), and Alzheimer’s disease (Sherman and Goldberg, 2001; Muchowski and Wacker, 2005), presumably by facilitating protein folding and inhibiting apoptosis (Leak, 2014). Genes enriched in multiple signaling pathways, like pathways of “Heterotrimeric G-protein signaling pathway” and “B cell activation,” were altered by Hspa1b deficiency in an MPTP-induced mouse model of Parkinson disease (Ban et al., 2012).

PRPF3 is one of several proteins interacting with U4 and U6 small nuclear ribonucleoproteins, which are components of spliceosomes. Mutations in *MECP2* (methyl-CpG-binding protein 2) cause the neurodevelopmental disorder Rett syndrome. The MECP2 protein directly interacts with PRPF3 (Long et al., 2011), and several Rett-associated mutations in *MECP2* affect interaction of MECP2 with PRPF3, implying that neurodevelopmental disorders, in general, could be related to abnormal mRNA splicing.

*SERPINA3* belongs to the serine protease inhibitor family. The protein antagonizes the activity of neutrophil cathepsin G and mast cell chymase and has been implicated in neuroinflammation, neurodegeneration (Baker et al., 2007), and other types of brain conditions such as human prion diseases (Vanni et al., 2017). The mRNA level of SERPINA3 is robustly up-regulated in the prefrontal cortex of schizophrenia patients, suggesting its involvement in the pathogenesis of neuropsychiatric disorders (Arion et al., 2007; Saetre et al., 2007; Fillman et al., 2014).

In summary, we identified genes that may function as ASD genetic loci targeted genes in the brain frontal cortex through multi-omics data analyses. These genes are worth being further characterized for their function in ASD development through experimental approaches.

## Author Contributions

YS was mainly involved in the data analysis, processing, and summarization. XY was involved in part of data analysis and mainly responsible for drafting manuscript. QX and JL were responsible for conception and design of study and revising the manuscript critically for important intellectual content. Other authors have partially participated in the work to take public responsibility for the content, including participation in the concept, design, analysis, writing, or revision of the manuscript.

## Funding

This study was supported by National Natural Science Foundation of China (81771769); Tianjin Natural Science Foundation (18JCYBJC42700); Startup Funding from Tianjin Medical University; and the Thousand Youth Talents Plan of Tianjin.

## Conflict of Interest Statement

The authors declare that the research was conducted in the absence of any commercial or financial relationships that could be construed as a potential conflict of interest.
