# Supplementary material for: Target Genes of Autism Risk Loci in Brain Frontal Cortex
Source: Front Genet. 2019 Aug 9;10:707. doi: 10.3389/fgene.2019.00707 (PMC6696877; doi:10.3389/fgene.2019.00707)
Supplement: Supplementary file 1 [file DataSheet_1.pdf]

Target Genes of Autism Risk Loci in Brain Frontal Cortex

Yan Sun, Xueming Yao, Michael E. March, Xinyi Meng, Junyi Li, Zhi Wei, Patrick M.A. Sleiman, Hakon Hakonarson, Qianghua Xia, Jin Li

Supplementary Material

Supplementary Table 1. Candidate ASD target genes that showed significant differential expression in microarray datasets.

ASD SNP	GWAS P-value	GWAS reference	Target gene	eQTL P-value	Microarray P-value
rs8321	8.00E-09	[1]	*ABCF1*	0.029	0.0379
rs2535629	3.00E-12	[1]	*ABHD14A*	0.015	0.0466
rs11191419	1.00E-16	[2]	*ACTR1A*	0.014	0.0249
rs8009147	2.00E-09	[2]	*ADSSL1*	0.0029	0.0315
rs1104918	6.00E-06	[1]	*AEN*	0.026	0.0176
rs6453278	5.00E-06	[3]	*AGGF1*	0.019	0.0444
rs11587682	7.00E-06	[1]	*APH1A*	0.037	0.0166
rs72687362	4.00E-09	[2]	*ARC*	0.013	0.0499
rs1080500	6.00E-09	[2]	*CACNA2D3*	0.0081	0.0334
rs4702	1.00E-10	[2]	*CIB1*	0.033	0.0388
rs115558405	2.00E-13	[2]	*CLIC1*	0.0018	0.0415
rs4773054	5.00E-08	[4]	*COL4A1*	0.0034	0.0117
rs12045323	7.00E-06	[5]	*DHRS3*	0.0092	0.0424
rs6071524	1.00E-09	[2]	*DHX35*	0.047	0.0343
rs12704290	1.00E-10	[2]	*DMTF1*	0.037	0.0443
rs11210892	4.00E-10	[2]	*DPH2*	0.0024	0.0315
rs7711337	8.00E-07	[6]	*GABRG2*	0.019	0.0315
rs171748	6.00E-09	[2]	*GNL3L*	0.048	0.0335
rs7184114	2.00E-08	[2]	*GPR56*	0.023	0.0173
rs2851447	8.00E-12	[2]	*HIP1R*	0.018	0.0315
rs75782365	8.00E-24	[2]	*HIST1H1C*	0.0015	0.0316
rs2021722	2.00E-12	[1]	*HLA-A*	0.022	0.0136
rs385492	4.00E-09	[2]	*HLA-F*	0.00097	0.002
rs4150167	3.00E-07	[6]	*HSDL1*	NaN	0.0477
rs3132581	2.00E-07	[1]	*HSPA1A*	0.0069	0.00247
rs3132581	2.00E-07	[1]	*HSPA1B*	0.0058	0.0121
rs548181	9.00E-07	[1]	*HYLS1*	0.041	0.0203
rs4905226	4.00E-06	[7]	*IFI27*	0.0011	0.0428
rs4650608	1.00E-06	[1]	*IFI44*	0.013	0.0315
rs1080500	6.00E-09	[2]	*IL17RB*	0.017	0.00776
rs17292804	6.00E-09	[2]	*INF2*	0.024	0.0401

rs2233375	4.00E-06	[8]	*INPP5D*	0.011	0.0104
rs609412	2.00E-06	[1]	*LARS*	0.015	0.0219
rs12871532	1.00E-06	[1]	*LIG4*	0.048	0.0445
rs6538761	2.00E-06	[9]	*LTA4H*	0.031	0.0314
rs11587682	7.00E-06	[1]	*MCL1*	0.011	0.0374
rs221902	4.00E-08	[2]	*MED6*	0.016	0.0303
rs73416724	3.00E-08	[2]	*MRPL2*	0.0067	0.0194
rs11587682	7.00E-06	[1]	*MTMR11*	0.026	0.0329
rs7700191	4.00E-06	[1]	*NFKB1*	0.015	0.0137
rs7254215	7.00E-06	[1]	*NOTCH3*	0.036	0.0344
rs1550976	2.00E-06	[8]	*NTM*	0.0099	0.0346
rs169738	8.00E-10	[2]	*NUDT3*	0.0036	0.0104
rs7914558	2.00E-09	[1]	*OBFC1*	0.0018	0.0172
rs760648	3.00E-08	[2]	*PACSIN2*	0.0055	0.0346
rs11735612	4.00E-06	[5]	*PCDH18*	0.005	0.0147
rs7746199	1.00E-20	[2]	*PGBD1*	0.017	0.0419
rs133047	1.00E-08	[2]	*PHF5A*	0.0082	0.0499
rs1797052	8.00E-09	[10]	*POLR3C*	0.03	0.0248
rs7700191	4.00E-06	[1]	*PPP3CA*	0.0018	0.0427
rs4702	1.00E-10	[2]	*PRC1*	0.01	0.0465
rs11587682	7.00E-06	[1]	*PRPF3*	0.017	0.0145
rs8054556	4.00E-10	[2]	*PRR14*	0.042	0.02
rs3849046	2.00E-09	[2]	*REEP2*	0.024	0.0359
rs2332700	2.00E-09	[2]	*RGS6*	0.024	0.042
rs116254153	4.00E-11	[2]	*RING1*	0.0015	0.0173
rs76994193	4.00E-08	[2]	*RPS6*	0.037	0.0349
rs1080500	6.00E-09	[2]	*SELK*	0.012	0.0285
rs4905226	4.00E-06	[7]	*SERPINA3*	0.014	0.0376
rs8054556	4.00E-10	[2]	*SEZ6L2*	0.014	0.0404
rs7914558	2.00E-09	[1]	*SH3PXD2A*	0.013	0.0277
rs12826178	4.00E-10	[2]	*SHMT2*	0.0084	0.0427
rs2898883	2.00E-08	[11]	*SPOP*	0.0081	0.0192
rs548181	9.00E-07	[1]	*SRPR*	0.035	0.0362
rs114882497	2.00E-16	[2]	*TAP1*	0.0012	0.0137
rs880446	4.00E-08	[2]	*TCEA2*	0.02	0.0319
rs2297909	2.00E-06	[1]	*TNNT2*	0.0099	0.0308
rs9607782	9.00E-09	[2]	*TNRC6B*	0.014	0.0474
rs2851447	8.00E-12	[2]	*VPS37B*	0.047	0.0499
rs72934570	1.00E-09	[2]	*WDR7*	0.047	0.0401
rs133047	1.00E-08	[2]	*XRCC6*	0.00082	0.0453
rs11210892	4.00E-10	[2]	*YBX1*	0.0013	0.0269
rs880446	4.00E-08	[2]	*ZBTB46*	0.035	0.0211
rs10255295	5.00E-06	[1]	*ZC3HAV1*	0.011	0.0315
rs12887734	2.00E-12	[2]	*ZFYVE21*	0.046	0.0226

rs56223946	3.00E-09	[2]	*ZNF395*	0.0097	0.0171

SNP = single nucleotide polymorphism; GWAS P-value = P-value of each ASD SNP in their original GWAS study; GWAS reference = reference for each ASD GWAS SNP; Target Gene = candidate target gene identified for each ASD GWAS SNP; eQTL P-value = P-value of correlation between gene expression level and GWAS SNP genotype in GTEx database or Braineac database; Microarray P-value = P-value of the differentially expressed genes in microarray meta-analysis.

Supplementary Table 2. Pathways significantly enriched among the 76 genes. The analyses were performed using web portal DAVID [12, 13] and PANTHER[14].

Web Portal	Pathway Database	Enriched Pathway	Count	Genes	P-value	Adjusted P-value
DAVID	KEGG	Antigen processing and presentation	5	HSPA1A, HSPA1B, HLA-A, HLA-F, TAP1	3.00E-04	3.60E-02
PANTHER	Reactome	Noncanonical activation of NOTCH3	3	APH1A, YBX1, NOTCH3	7.53E-06	1.65E-02

Count = The number of potential ASD target genes involved in each pathway; P-value = nominal P-value; Adjusted P-value = P-value after multiple testing adjustment.

Supplementary Figure 1. The ASD SNPs and target genes locate in same topologically associating domain (TAD) as reported in brain Hi-C data.

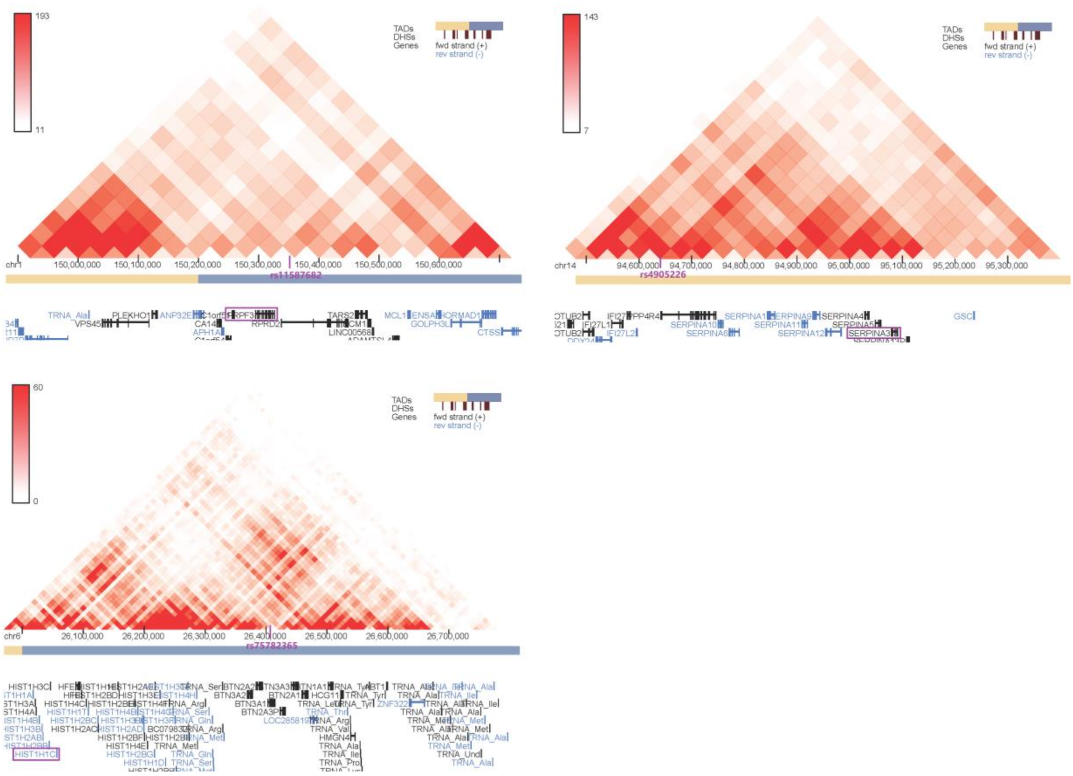

Reference

1. *Identification of risk loci with shared effects on five major psychiatric disorders: a genome-wide analysis.* Lancet, 2013. **381**(9875): p. 1371-1379.
2. *Meta-analysis of GWAS of over 16,000 individuals with autism spectrum disorder highlights a novel locus at 10q24.32 and a significant overlap with schizophrenia.* Mol Autism, 2017. **8**(21): p. 017-0137.
3. Weiss, L.A., et al., *A genome-wide linkage and association scan reveals novel loci for autism.* Nature, 2009. **461**(7265): p. 802-8.
4. Chaste, P., et al., *A genome-wide association study of autism using the Simons Simplex Collection: Does reducing phenotypic heterogeneity in autism increase genetic homogeneity?* Biol Psychiatry, 2015. **77**(9): p. 775-84.
5. Guo, W., et al., *Polygenic risk score and heritability estimates reveals a genetic relationship between ASD and OCD.* Eur Neuropsychopharmacol, 2017. **27**(7): p. 657-666.
6. Anney, R., et al., *Individual common variants exert weak effects on the risk for autism spectrum disorders.* Hum Mol Genet, 2012. **21**(21): p. 4781-92.
7. St Pourcain, B., et al., *Variability in the common genetic architecture of social-communication spectrum phenotypes during childhood and adolescence.* Mol Autism, 2014. **5**(1): p. 2040-2392.
8. Salyakina, D., et al., *Variants in several genomic regions associated with asperger disorder.* Autism Res, 2010. **3**(6): p. 303-10.
9. Xia, K., et al., *Common genetic variants on 1p13.2 associate with risk of autism.* Mol Psychiatry, 2014. **19**(11): p. 1212-9.
10. Goodbourn, P.T., et al., *Variants in the 1q21 risk region are associated with a visual endophenotype of autism and schizophrenia.* Genes Brain Behav, 2014. **13**(2): p. 144-51.
11. Cantor, R.M., et al., *ASD restricted and repetitive behaviors associated at 17q21.33: genes prioritized by expression in fetal brains.* Mol Psychiatry, 2018. **23**(4): p. 993-1000.
12. Huang da, W., B.T. Sherman, and R.A. Lempicki, *Systematic and integrative analysis of large gene lists using DAVID bioinformatics resources.* Nat Protoc, 2009. **4**(1): p. 44-57.
13. Huang da, W., B.T. Sherman, and R.A. Lempicki, *Bioinformatics enrichment tools: paths toward the comprehensive functional analysis of large gene lists.* Nucleic Acids Res, 2009. **37**(1): p. 1-13.
14. Mi, H. and P. Thomas, *PANTHER pathway: an ontology-based pathway database coupled with data analysis tools.* Methods Mol Biol, 2009. **563**: p. 123-40.
